# The importance of structural softening for the evolution and architecture of passive margins

**DOI:** 10.1038/srep38704

**Published:** 2016-12-08

**Authors:** T. Duretz, B. Petri, G. Mohn, S. M. Schmalholz, F. L. Schenker, O. Müntener

**Affiliations:** 1 Institute of Earth Sciences, University of Lausanne, Géopolis, CH-1015 Lausanne, Switzerland; 2 École et Observatoire des Sciences de la Terre, Institut de Physique du Globe de Strasbourg – CNRS UMR7516, Université de Strasbourg, 1 rue Blessig, F−67084, Strasbourg Cedex, France; 3 Département Géosciences et Environnement, Université de Cergy-Pontoise, 5, mail Gay Lussac, Neuville-sur-Oise, 95031 Cergy-Pontoise Cedex, France; 4 Institute of Earth Sciences, University of Applied Sciences and Arts of Southern Switzerland (SUPSI), Via Trevano, CH-6952 Canobbio, Switzerland.

## Abstract

Lithospheric extension can generate passive margins that bound oceans worldwide. Detailed geological and geophysical studies in present and fossil passive margins have highlighted the complexity of their architecture and their multi-stage deformation history. Previous modeling studies have shown the significant impact of coarse mechanical layering of the lithosphere (2 to 4 layer crust and mantle) on passive margin formation. We built upon these studies and design high-resolution (~100–300 m) thermo-mechanical numerical models that incorporate finer mechanical layering (kilometer scale) mimicking tectonically inherited heterogeneities. During lithospheric extension a variety of extensional structures arises naturally due to (1) structural softening caused by necking of mechanically strong layers and (2) the establishment of a network of weak layers across the deforming multi-layered lithosphere. We argue that structural softening in a multi-layered lithosphere is the main cause for the observed multi-stage evolution and architecture of magma-poor passive margins.

Passive continental margins are the focus of intense research activity since decades^1^,^2^,^3^, enhanced by the intensification of both industrial and academic geophysical surveys associated with ocean drilling programs (ODP). These research programs brought evidence of hyper-extended domains associated with severely thinned crust (<10 km thick), exhumed subcontinental mantle and crustal allochthons^4^. These observations led to new conceptual and mechanical models for the formation of rift basins and magma-poor margins^5^,^6^. Rifting eventually leading to continental break-up develops in lithospheres that exhibit a complex pre-rift evolution characterized especially by orogenic to post-orogenic processes^7^. For example, Mesozoic rifting in Europe occurred in a lithosphere affected by the Variscan orogeny^8^, while Northern Atlantic rifting took place in a lithosphere affected by the Caledonian orogeny^9^ and South China Sea rifting succeeded the Yenshanian orogeny^10^. During these pre-rift events, interaction of magmatic, metamorphic and tectonic processes generates a heterogeneous continental lithosphere (Fig. 1a). The mechanical heterogeneity derived from (i) mechanical anisotropies^11^,^12^, (ii) a layered crust with a variably metamorphosed upper crust, a migmatitic middle crust and a granulitic lower crust^13^ sometimes hosting underplated mafic plutons^14^,^15^ occasionally associated with shallower granitic plutons^16^, and (iii) a layered mantle with structural (e.g. shear zones) or compositional remnants (e.g. pyroxenite) from ancient collisional events^17^. Such tectonic inheritance results in an overall layered continental lithosphere^18^,^19^ where mechanical heterogeneities range from a few meters to several kilometers in thickness (Fig. 1a).

The extension of small-scale power-law viscous or visco-plastic layered materials can result in shear localization and asymmetric extension^20^. This behavior is the result of structural softening due to necking of strong layers and formation of a network of interconnected weak layers. Structural softening is hence caused by internal reorganizations within heterogeneous materials in order to minimize deformational work^21^. Structural softening differs from material softening that requires an *a priori* defined alteration of material properties with increasing strain and which has been implemented in numerical models to explain the structures of rifted margins^22^. The role of coarse mechanical layering of the lithosphere, inherited dipping heterogeneities, and the role of bimineralic crust and mantle on rifting processes has been addressed in previous studies^5^,^23^,^24^,^25^,^26^,^27^,^28^,^29^,^30^,^31^. Here we present high-resolution numerical models that incorporate layering at finer scale, which show that structural softening arises during the extension of lithosphere and controls the development of the key structures observed in passive margins.

The numerical algorithm is based on the finite-difference marker-in-cell approach and incorporates gravity, visco-plasticity, temperature- and stress-dependent rheologies (Table 1), heat transfer and radiogenic heat production including shear heating and a newly developed free surface approach^32^. The initial heterogeneities in the continental lithosphere are approximated by an alternation of mechanically stronger and weaker layers of various dimensions (Fig. 1b).

We have tested different initial mechanical heterogeneities by varying the number of strong layers in both the lithospheric mantle and continental crust. Depending on the initial layering, the evolution and final architecture of passive margins change drastically. In the model devoid of layering within the crust and mantle lithosphere (Fig. 2a), the entire lithosphere deforms by single-phase necking with the development of a symmetric set of conjugate margins^33^,^27^. The general architecture displays narrow margins where the transition from a 30 km thick continental crust to a zone of exhumed mantle occurs over a few tens of kilometers what corresponds to the narrow rift mode^34^. In the model with five strong layers distributed across the crust and mantle lithosphere (each 5 km thick, Fig. 2b) the final architecture of the margins changes significantly. The deformation of crust and mantle (t = 3.9 Ma) is mechanically decoupled and is localized at different horizontal locations while the overall extension is symmetric. In a second stage (from t = 8.6 Ma), the crust records a general pure-shear deformation accommodated by the weak layers. Mantle upwelling occurs along one large-offset extensional shear zone depicting a general asymmetric architecture to the rift system. Eventually a wide region (>100 km) of strongly extended crust constitutes the distal domain, which does not reach a stage of subcontinental mantle exhumation. The residual weak continental crust preserved strong crustal lenses surrounded by weaker crust associated with undulations of the Moho. This architecture is comparable with the South China Sea margin^35^. In the model with eleven strong layers (each 5 km thick, Fig. 2c), the lithosphere also undergoes two-stage rifting. The deformation is increasingly localized and structural features such as detachments and necking zones (Fig. 2) are more pronounced. In addition, after 10.2 Ma the lithospheric mantle is exhumed at the seafloor.

These results illustrate the dramatic impact of structural softening during extension of a heterogeneous lithosphere. This mechanism controls: (1) the width of the necking zone and distal domains (narrow *vs.* wide) (2) the dominant deformation mode (pure shear-like *vs.* simple shear-like) and (3) the general geometry of the margin (symmetric *vs.* asymmetric).

A detailed analysis of a simulation with nine strong layers illustrates the multi-stage evolution of a passive margin (Fig. 3). In the first thinning stage (Fig. 3a), extension of the lithosphere is overall symmetric although crust and mantle exhibit contrasting deformation styles. Upper crustal levels deform in the brittle-plastic regime generating two conjugate sets of extensional shear zones and forming a graben. In the lower crust, the competent layers undergo necking, which generates zones of intense ductile deformation in agreement with interpretation of seismic reflection data offshore Britain (SWAT 1 section)^36^ or inferred from the remnants of the Alpine Tethys margins^37^. With progressive extension (t = 6.8 Ma, Fig. 3b), individual strong layers at different depths are disconnected due to necking and can subsequently be horizontally separated by distances reaching 10 to 50 km. The separating strong layers are thereby extracted from between their over- and underlying weak layers. Connections of such extraction zones form an anastomosing network of shear zones within weak layers that act as décollement horizons during vertical thinning. Thus, extreme lithospheric thinning results from the interaction of large-offset extensional shear zones that cut across strong layers extracting mid-crustal layers from the future distal margin (e.g. extraction zone). Lateral extraction of mid-crustal layers results in the formation of hyper-extended domains, which are characterized by the juxtaposition of upper and lower crustal layers. Similar mechanical extraction zones are active in the mantle but the overall necking occurs along two convex-upward large-offset shear zones.

Further extension (Fig. 3c, t = 10.3 Ma) amplifies the interaction of necking zones and couples the large-offset shear zones in the mantle with the crustal low-angle faults generating a major lithospheric detachment fault. This detachment fault generates overall asymmetric rifting (Fig. 3c). The dominant detachment zone accommodates large vertical and horizontal displacements, eventually leading to crustal breakup and subcontinental mantle exhumation. Deep crustal levels and subcontinental mantle are exhumed and emplaced in the distal regions of the two margins. During the latest stages of lithospheric thinning prior to breakup, shearing along the detachment fault detaches an overlying crustal block that forms an isolated upper crustal allochthon.

The results illustrate that lithospheric thinning is the result of a complex interplay between different extensional structures: (1) high-angle normal faults associated with tilted blocks cutting across strong layers and rooting in weak layers, (2) necking and boudinage of competent layers, (3) vertical ductile thinning of weak layers, (4) development of anastomosing shear zones in weak layers acting as décollement levels and accommodating the lateral extraction of strong layers, (5) low-angle detachment faults exhuming the subcontinental mantle and accompanying the formation of crustal allochthons. Notably, most of these structures have been identified on land^38^,^39^ and offshore^35^,^40^,^41^, although their significance and interplay are debated. These structures accommodate overall necking of the continental lithosphere by extraction of strong mid-crustal layers from the distal margin, leaving remnants of strong upper and lower crustal layers as observed in the Alpine Tethys^38^. This extraction mechanism explains the counterintuitive observation that hyper-extended and distal domains of some margins are composed of strong crustal rocks, which lack a significant syn-rift ductile deformation (e.g. the fossil Tasna Ocean-Continent Transition in the Alps)^42^. As reported for the Iberia-Newfoundland margins^43^,^44^, only the late rifting stages show a significant removal of weak material triggering the mechanical coupling of the residual crust and mantle lithosphere. Formation and emplacement of crustal allochthons on top of subcontinental mantle exhuming to the seafloor occurs just before crustal breakup.

Mechanical layering of the crust and mantle lithosphere triggers structural instabilities and structural softening during lithospheric extension. The model results highlight the key role of strong layers and especially of the lower-most strong crustal layer that undergoes necking first and further allows mechanical connection of weak crustal and mantle layers. Although our models do not consider fluid- and melting-related processes and mineralogical transformations, they provide first order geologically sound rift evolution scenarios. We suggest that passive margin formation is controlled by multilayer necking, extraction and symmetry-breaking shear zones. The generation of structures and intensity of shear localization depends on the degree of tectonic inheritance (i.e. number of layers, strength contrast, spacing). We suggest that the variability of passive margin architecture (e.g. fossil Alpine Tethys, South-China Sea, Iberia-Newfoundland, South-Atlantic margins) is largely caused by differences in pre-rift lithospheric structures, which in turn induces contrasting structural instabilities and related softening. These results further indicate that if kilometer-scale heterogeneities and related structural softening are numerically resolved then predefined material strain softening is not further essential to model the evolution and architecture of passive margins. Modeling structural instabilities and related softening during rifting requires the numerical resolution of the length-scale of tectonically inherited heterogeneities. With increasing computational power, future models will allow resolving even finer structures and associated softening, in the aim of further refining our knowledge about passive margin forming processes.

## Methods

The applied numerical algorithm solves the equations of momentum, mass and energy conservation based on the Finite-Difference/Marker-in-Cell method^45^. The linear momentum equation takes the form of


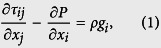


where *τ*_*ij*_ and *P* respectively corresponds to the deviatoric stress tensor and the pressure.

We use the Boussinesq approximation, the materials are hence assumed incompressible and the mass conservation equation is formulated as


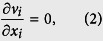


where *v*_*i*_ corresponds to the components of the velocity vector. The energy conservation equation takes into account the effect of viscous dissipation and radioactive heat production and is written as


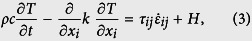


where *T* is the temperature, *ρ* is the density, *c* is the heat capacity, *k* is the thermal conductivity, *H* is the radioactive heat productivity and 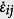 is the deviatoric strain rate tensor. There are no advection terms in the energy conservation equation because temperatures are advected with marker points (see below).

We employ a grid resolution of 1001*801 (horizontal*vertical direction) nodes with initially 4*4 markers per cell. The markers are advected using a 1^st^ order in time/4^th^ order in space Runge-Kutta scheme. In order to avoid spurious instabilities inherent to the explicit advection scheme, we use a free surface stabilization scheme^46^. Impact of the numerical resolution on the modeling results is depicted in Supplementary Fig. 1.

The model domain has and initial width of 300 km and depth of 120 km. The initial crustal thickness is set to 35 km and the mantle lithosphere extends down to 70 km. All models were run with a constant bulk extension rate (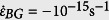). The velocity component normal to each boundary (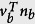) is calculated at each time step according to 
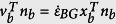
, where *n*_*b*_ and *x*_*b*_ are the normal unit of the boundary *b* and the coordinate vectors of the boundary nodes. The domain boundaries are advected at each time step using the corresponding velocity such that the volume of the domain is conserved (no addition and no removal of material). Free slip is imposed at the left, right and bottom boundaries while the top boundary is a free surface. The thermal structure is initialized assuming an equilibrated thermal state using the parameters of Table 1. The top and bottom temperatures are respectively set to 0 and 1350 °C and the left and right sides exhibit zero heat flux. The influence of the presence of an additional layer of adiabatic mantle representing the asthenosphere is depicted in Supplementary Fig. 2.

The material densities depend on both the pressure and temperature according to


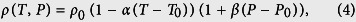


where *α* is set to 8 × 10^−6^ K^−1^, *β* is 1 × 10^−11^ Pa^−1^, *T*_*0*_ is 273 K and *P*_*0*_ is 0 Pa. The viscosities are calculated according to a dislocation creep flow law.


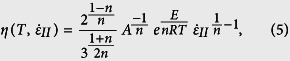


where 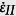 is the second invariant of the strain rate tensor and the remaining symbols are defined in Table 1. The mechanically strong crustal layers deform according to a felsic granulite flow law^47^, the weak crustal layers have wet quartzite rheology^48^, the strong mantle layers have a dry olivine flow law and the weak mantle has a wet olivine rheology^49^. The alternation of layers characterized by wet and dry olivine rheology is a pragmatic way to achieve mechanical layering in the lithospheric mantle (i.e. it does not imply any stratification in its degree of hydration).

If the effective viscous stress exceeds the Drucker-Prager yield stress, an effective visco-plastic viscosity is substituted to the viscosity,


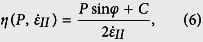


where *P* corresponds to the pressure (including gravity). We do not include a predefined material strain softening (e.g. reduction of friction coefficient or effective viscosity with progressive strain) nor the energetics and rheological impact of asthenospheric melting. Initial random noise is applied at each layers interface by imposing a random offset of vertical marker location with maximum amplitude of 500 m.

## Additional Information

**How to cite this article**: Duretz, T. *et al*. The importance of structural softening for the evolution and architecture of passive margins. *Sci. Rep.* **6**, 38704; doi: 10.1038/srep38704 (2016).

**Publisher's note:** Springer Nature remains neutral with regard to jurisdictional claims in published maps and institutional affiliations.

## Supplementary Material

Supplementary Information

## Figures and Tables

**Figure 1:**
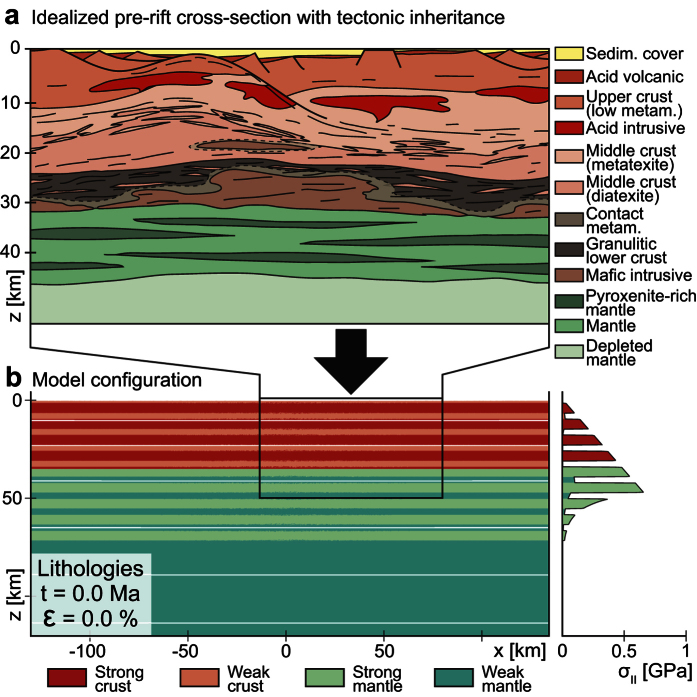
(**a**) Heterogeneous pre-rift lithosphere inspired from the pre-Mesozoic rifting structure of the European lithosphere that underwent Variscan and post-Variscan processes. (**b**) Idealized model configuration (enlargement), mechanical heterogeneities due to tectonic inheritance are represented with layering. The lower right panel depicts the corresponding idealized rheological profile.

**Figure 2:**
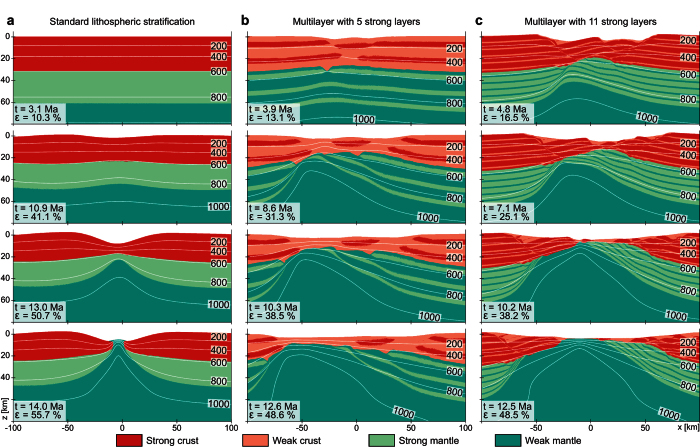
Numerical models of the evolution of rifting incorporating (**a**) a basic lithospheric structure, (**a**) 5 competent layers (**b**,**c**) and 11 competent layers. The color scale corresponds to enlargements of the different lithologies and the contour lines represent the isotherms in degree Celsius.

**Figure 3:**
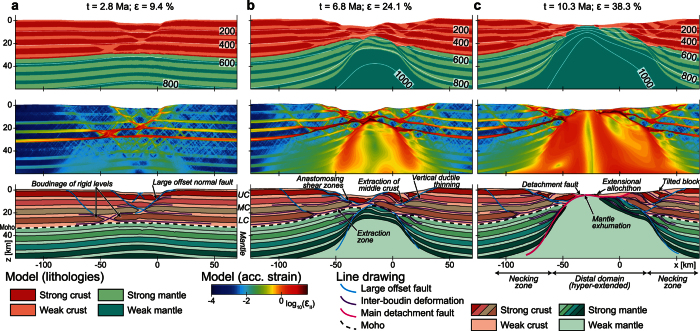
Time evolution of a model incorporating 9 competent layers. Uppermost panels represent enlargements of the lithological distribution. The mid panels correspond to enlargements of the accumulated von Mises visco-plastic strain (ε_II_). Lowermost panels depict line drawings and geological interpretation of model results, which emphasize the main features that develop throughout the evolution of the models. Panel (a) corresponds to the initial extension phase during which crust and mantle are decoupled (9.4% of extension), contour lines represent the isotherms. Panel (b) shows the geometry of the model after 24.1% of extension. At this stage, deformation of the crust and mantle is coupled. Panel (c) corresponds to the final rifting stage involving mantle exhumation (38.3% of extension).

**Table 1:** Thermal and rheological parameters used in the simulations.

	*H* [W.m^−3^]	*k* [W.m^−1^.K^−1^]	*ρ*_*0*_ [kg.m^−3^]	*A* [Pa^−n^.s^−1^]	*E* [kJ.mol^−1^]	*n*	*Φ*	*C* [MPa]
S. C.	5 × 10^−7^	2.5	2800	5.0477 × 10^−28^	485	4.7	30	10
W. C	5 × 10^−7^	2.5	2800	5.0717 × 10^−18^	154	2.3	5	1
S. M.	1 × 10^−10^	3.0	3250	2.5519 × 10^−17^	532	3.5	30	10
W. M.	1 × 10^−10^	3.0	3250	1.9953 × 10^−21^	471	4.0	5	1

S. C., W. C., S. M. and W. M. respectively stand for mechanically strong crust, weak crust, strong mantle and weak mantle. The symbols *H, k, ρ*_*0*_, *A, E, n, Φ* and *C* correspond to the radiogenic heat productivity, thermal conductivity, reference density, flow law pre-exponent, activation energy, stress exponent, friction angle and cohesion. The heat capacity has a constant value of 1050 J/kg/K for all lithologies.
