# Supplementary material for: The importance of structural softening for the evolution and architecture of passive margins
Source: Sci Rep. 2016 Dec 8;6:38704. doi: 10.1038/srep38704 (PMC5144128; doi:10.1038/srep38704)
Supplement: Supplementary Information [file srep38704-s1.doc]

**Supplementary Information**

THE IMPORTANCE OF STRUCTURAL SOFTENING FOR THE EVOLUTION AND ARCHITECTURE OF PASSIVE MARGINS

Thibault Duretz, Benoit Petri, Geoffroy Mohn, Stefan M. Schmalholz, Filippo L. Schenker and Othmar Müntener.

**SUPPLEMENTARY FIGURES**

**Supplementary Figure 1**

Impact of the numerical resolution on the modeling results. The colors correspond to the different lithologies after 25 % extension (enlargements). The medium-resolution run (501*401 nodes, panel b), the reference run (1001*801 nodes c) and the high-resolution run (2001*1601 nodes c) exhibit similar dynamics. The low-resolution simulation (251*201 nodes, panel a) is characterised by more distributed deformation. Although the models were run with different initial random noise (inherent to the resolution), qualitatively good numerical convergence is obtained with increasing resolution.


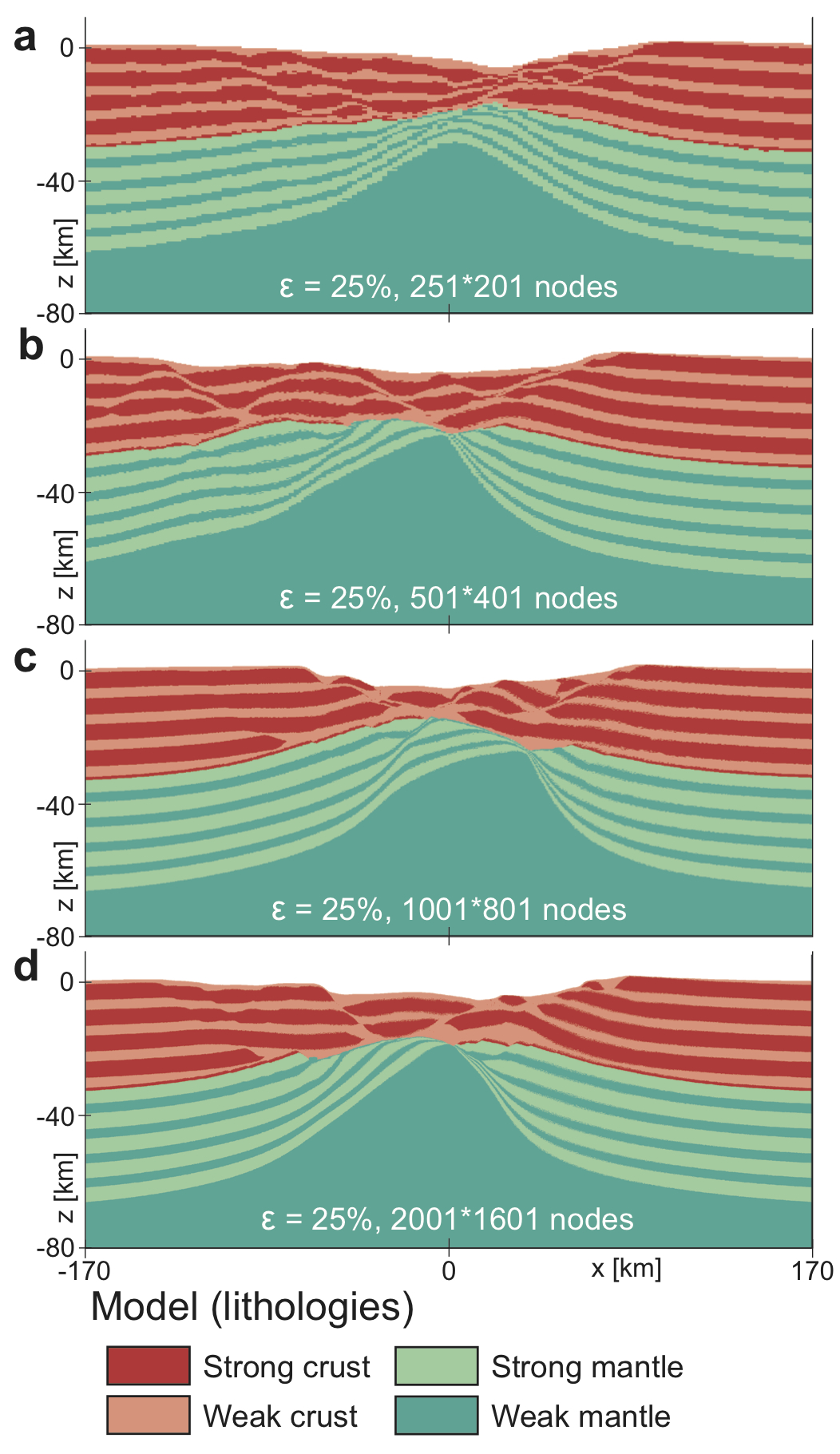


**Supplementary Figure 2**

Evolution of a numerical model using larger model domain (initially 300*300 km with a resolution of 1001*1601 nodes) and encompassing an additional layer of asthenosphere. The adiabatic mantle has the same rheology than the Weak mantle (cf. Table 1) but is characterised by an unrealistically large thermal conductivity (3000 W/m/K) hence mimicking an adiabatic behaviour. The panels depicts enlargement of the lithological field for different amounts of extension (10 %, panel a; 25 % panel b; 40 %, panel c). All models parameters are kept the same than in the reference model, except for the initial perturbation.

The model exhibits a similar time evolution than the reference model and involves necking of strong layers, extraction of strong layers, interconnection of weak phases and has a similar timing of breakup. The model thickness and presence of an asthenospheric layer hence has a moderate impact on our reference model results.

**
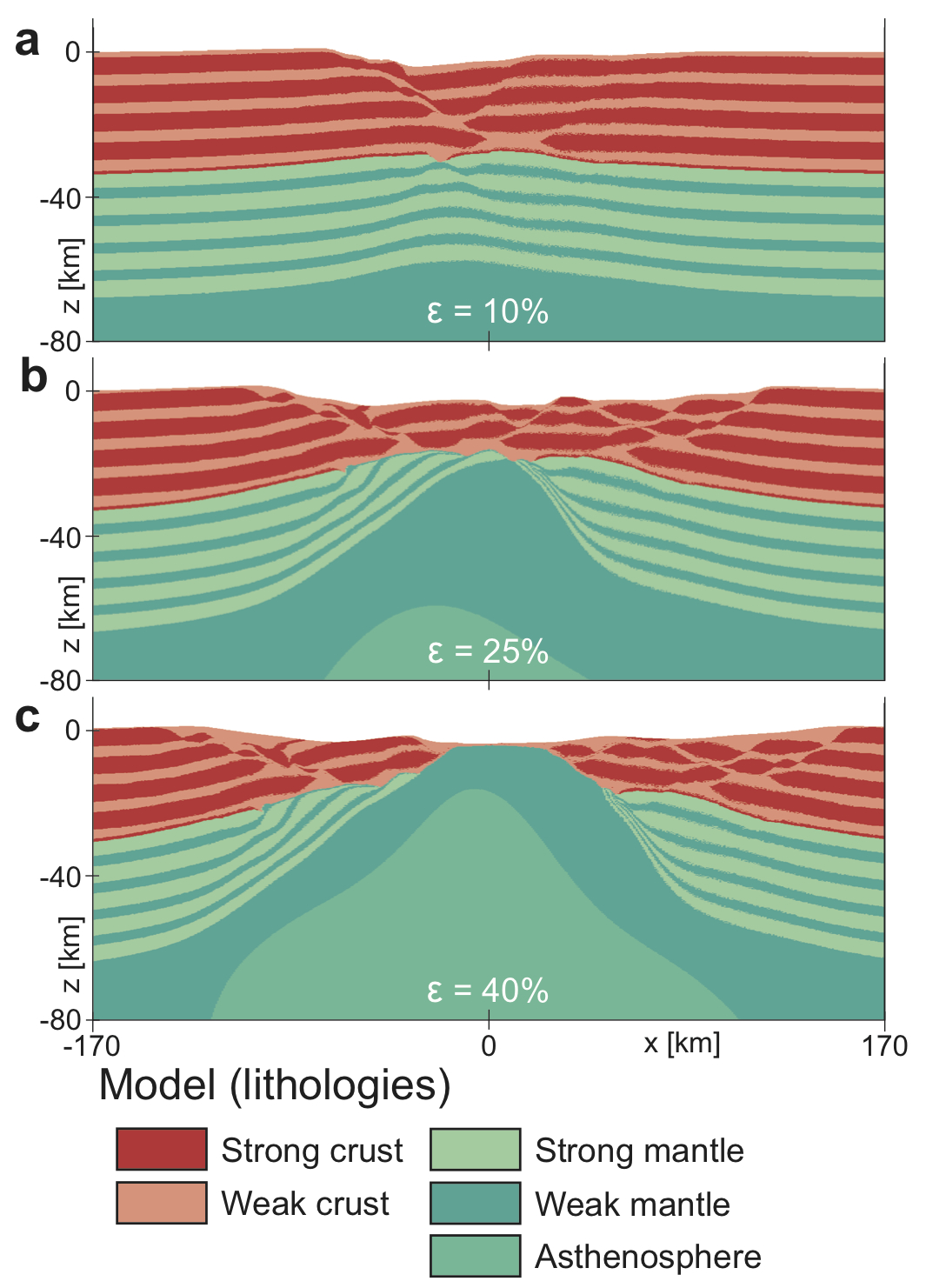
**
